# An Image Augmentation Method Based on Limited Samples for Object Tracking Based on Mobile Platform

**DOI:** 10.3390/s22051967

**Published:** 2022-03-02

**Authors:** Zihao Wang, Sen Yang, Mengji Shi, Kaiyu Qin

**Affiliations:** 1 School of Aeronautics and Astronautics, University of Electronic Science and Technology of China, Chengdu 611731, China; 201611191002@std.uestc.edu.cn (Z.W.); 2018100402009@std.uestc.edu.cn (S.Y.); maangat@uestc.edu.cn (M.S.); 2 Aircraft Swarm Intelligent Sensing and Cooperative Control Key Laboratory of Sichuan Province, Chengdu 611731, China

**Keywords:** real-time object tracking, limited samples, projection augmentation, blur augmentation, monocular vision

## Abstract

This paper proposes an image augmentation model of limited samples on the mobile platform for object tracking. The augmentation method mainly aims at the detection failure caused by the small number of effective samples, jitter of tracking platform, and relative rotation between camera and object in the tracking process. Aiming at the object tracking problem, we first propose to use geometric projection transformation, multi-directional overlay blurring, and random background filling to improve the generalization ability of samples. Then, selecting suitable traditional augmentation methods as the supplements, an image augmentation model with an adjustable probability factor is provided to simulate various kinds of samples to help the detection model carry out more reliable training. Finally, combined with a spatial localization algorithm based on geometric constraints proposed by the author’s previous work, a framework for object tracking with an image augmentation method is proposed. SSD, YOLOv3, YOLOv4, and YOLOx are adopted in the experiment of this paper as the detection models. And a large number of object recognition and object tracking experiments are carried out by combining with common data sets OTB50 and OTB100 as well as the OTMP data set proposed by us for mobile platform. The augmented module proposed in this paper is conducive for the detection model to improve the detection accuracy by at least 10%. Especially for objects with planar characteristics, the affine and projection transformation used in this paper can greatly improve the detection accuracy of the model. Based on the object tracking framework of our augmented model, the RMSE is estimated to be less than 4.21 cm in terms of the actual tracking of indoor objects.

## 1. Introduction

With the rapid development of deep learning theory in the field of computer vision, image data samples, as one of the key core driving forces in various learning models, play a decisive role in neural network model training. Especially in the task of target recognition and object tracking, improving the quality and quantity of samples can play a decisive role in the final object recognition and tracking. Therefore, as an important means, image augmentation technology can generate “new” image sample data sets through various types of transformation processing of image data. The function is to expand the number of sample sets that can be trained by the network and improve the model generalization ability. The Lenet-5 model [1] proposed by LeCun et al., in 1998 put forward the similarity transformation and affine transformation of image samples to propose the generalization ability of the model. In recent years, with the rapid development and evolution of convolutional neural networks, a large number of neural network models are often required to carry out various image transformations and enlargement in the pre-processing stage of samples. In 2012, Krizhevsky et al., proposed AlexNet [2], a color space enhancement algorithm based on Principal Component Analysis (PCA) that carried out sample cutting and mirror transformation to make the training data set be able to summarize the characteristics of samples more comprehensively. The VGG [3] network proposed by Simon-Yan et al., in 2014 used multi-scale scaling and clipping to carry out image augmentation. In GoogLeNet [4] proposed by Szegedy et al., in 2015, the method of sample clipping and mirroring in AlexNet was further extended. In the test, an image sample was expanded to 144 samples, and Softmax probability values of all samples were averaged for related applications of image classification. The residual network (ResNet) [5] proposed by He et al., in 2016 and the densely connected convolutional networks (DenseNet) [6] proposed by Huang et al., in 2017 also adopt the method of two-dimensional geometric transformation to carry out image data augmentation, and the accuracy was significantly achieved. In 2017, Z Hussain et al., explained that image enhancement methods need to be constructed in combination with specific scientific tasks in specific medical scenarios based on rotation, translation, principal component analysis, and other augmentation methods [7]. Bochkovskiy et al., added Cutmix [8] and Mosaic to YOLOv4 [9] proposed in 2020 for image augmentation and adopted self-adversarial training that reduces the negative effect of small datasets. Image augmentation methods such as Mixup [10] and Mosaic are used in YOLOx [11] proposed by Zheng Ge et al., in 2021. Zhun Zhong et al., proposed a random erasing image augmentation method in 2020, which Random Erasing randomly selects a rectangle region in an image and erases its pixels with random values [12]. Researchers have also studied the use of multiple image combinations for information mixing, sampling pair augmentation proposed by Inoue et al. [13]. Since then, with the emergence of new deep learning concepts such as reinforcement learning [14] and transfer learning [15], the idea of using neural network models to generate new image samples has been proposed by researchers. Augment [16] and Rand Augment [17], etc., the method with automatic searching here provides a new idea for image augmentation.

However, it is worth noting that most of the augmentation methods mentioned above process images based on 2D images. Although Michael Niemeyer et al., proposed a very excellent and effective 3D projection augmentation method called GIRAFFE in 2021 [18]. However, the method is based on the massive data, and the time cost and resource cost required for augmentation are huge. In fact, in many object tracking tasks, especially for non-cooperative object tracking, it is often difficult for us to obtain a large number of trainable samples in the pre-processing stage. Based on the above consideration, we propose a data augmentation method for a small sample set, which can be used to deal with 3D object tracking tasks on the moving platform. The main innovations of this paper are as follows:

1. A multi-directional stacking blur augmentation method is proposed to deal with the imaging blur problem caused by the platform shaking and the fast-moving object in the scene of a mobile platform. Experiments show that the blur augmentation method can effectively improve the recognition accuracy of the test set with imaging blur characteristics. A random background padding method is proposed to deal with the missing images such as black borders and black blocks in the augmented area due to geometric transformation. Experiments show that the background padding method can improve the recognition accuracy of the neural-network-based detection model to a certain extent.
2. Based on the two augmentation methods in part 1, combined with traditional augmentation methods such as geometric augmentation, brightness adjustment, Gaussian noise injection, and color jittering, an image augmentation model is proposed to deal with the problem of object tracking in insufficient samples. Experiments show that the model can effectively improve the tracking accuracy of random moving objects in three-dimensional space.
3. Combined with the author’s previous research work on object tracking [19,20], the object localization and tracking framework with image augmentation for limited samples is proposed. This framework can effectively deal with the problem of object loss caused by space rotation, platform jitter, and fast movement of the object in the 3D tracking. Besides, we use multiple open-source datasets for testing, which verifies the reliability and stability of the algorithm proposed in this paper.

The rest of this paper is organized as follows. In Section 2, the preparatory research related to the content of this paper is introduced. Section 3 introduces the image augmentation model proposed in this paper in detail, and combined with the positioning algorithm based on geometric constraints, a motion platform-based 3D object tracking framework with Limited Samples Augmentation is proposed. Section 4 carries out experiments based on multiple open data sets and detailed data analysis is given. In Section 5, a summary is made to conclude the paper.

## 2. Related Works

### 2.1. Datasets for Tracking

In addition to using the common object tracking data sets such as OTB50 [21] and OTB100 [22], this paper also provides a Benchmark for Object Tracking with Motion Parameters (OTMP) designed by us. OTMP data set gives images obtained by the monocular camera, the motion trajectory of the monocular camera, the motion trajectory of the samples of the object to be trained, as well as camera internal parameters. The OTMP data set designed in our paper aims to provide an indoor data set with camera motion and 3D motion trajectory in template space, as well as experimental simulation and verification basis for sample training with depth parameters, visual slam, 3D positioning, and tracking problems. We have uploaded the OTMP to GitHub: https://github.com/6wa-car/OTMP-DataSet.git (accessed on 30 December 2021).

### 2.2. SPPM for 3D Positioning Method

**Figure 1:**
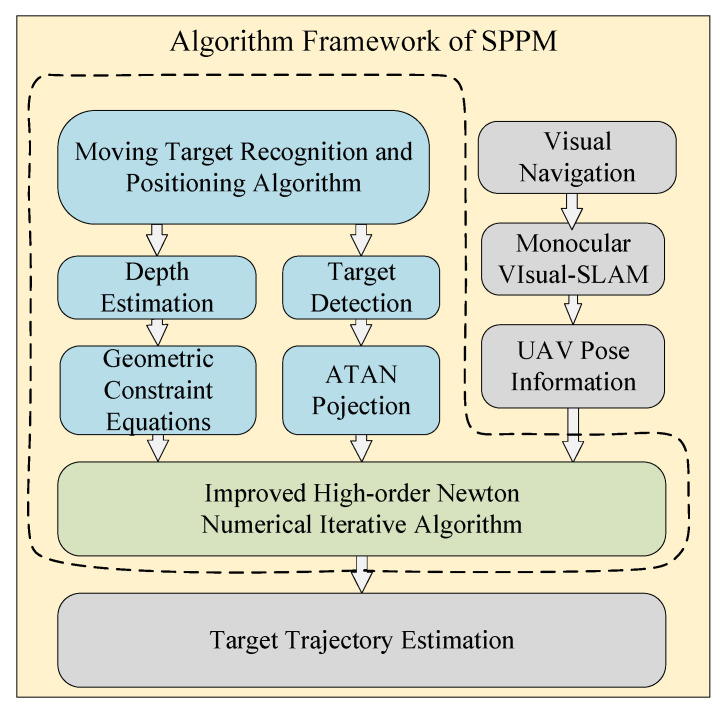
Schematic diagram of SPPM framework.

The Single-frame Parallel-features Positioning Method (SPPM) [19] aims to solve the problem of tracking dynamic objects, and it extracts the coplanar parallel constraint relations between object feature points to construct high-order nonlinear over-determined equations with unknown depth values. Then it introduces an improved Newton numerical optimization based on the Runge-Kutta method [23], which greatly reduces the error caused by 2D detection. Figure 1 shows the core algorithm of SPPM:

The main function of the SPPM is to solve the spatial depth of the object. The depth information solution also called the scale calculation problem, is one of the key problems in the object positioning and tracking technology based on monocular vision. In this paper, the camera projection model and geometric constraint equations are used to construct the depth equations. The object is abstracted as a rectangle or a parallelogram, and the improved high-order Newton iterative algorithm is used to realize the efficient real-time numerical solution of the depth information of the object feature points. Finally, the Kalman filter and linear regression are used to filter and estimate the object trajectory.

In Section 3.6, we combine the proposed augmented method with the spatial positioning and tracking algorithm to propose a 3D object tracking algorithm framework based on the moving platform.

### 2.3. Detection Model and Augmentation Methods

Due to the high real-time requirements of object tracking tasks, the two algorithms used for the test of this paper are single-stage methods, including YOLOv3 [24] and Single Shot MultiBox Detector (SSD) [25]. YOLOv1 (You Only Look Once) [26] was first proposed by Joseph Redmon et al., in 2015, and it generates candidate boxes through sliding windows and only adopts a single CNN model to achieve end-to-end target detection with high computing speed. YOLOv3 is an upgraded version proposed in 2018. SSD algorithm was proposed by Wei Liu et al., in 2016, and it utilizes multi-layer scale detection and combines Yolo’s regression idea with Faster R-CNN’s [27] anchor box mechanism, which improves accuracy on the premise of guaranteeing the speed.

It is worth noting that SSD adopts a series of image enhancement methods such as brightness, saturation, hue, and clipping. On the other hand, YOLO series models such as YOLOv4 [9] and YOLOx [11] add new image augmentation methods such as Mixup, Cutmix, and Mosaic, and involve self-adversarial training that reduces the negative effect of small datasets.

However, they don’t solve the problems well such as spatial rotation and jittering in the object tracking, which are also the main problems to be solved by our augmented method in this paper. Table 1 illustrates how our method differs from previous augmentation methods:

## 3. Image Augmentation Based on Limited Samples

### 3.1. Overall Framework

**Figure 2:**
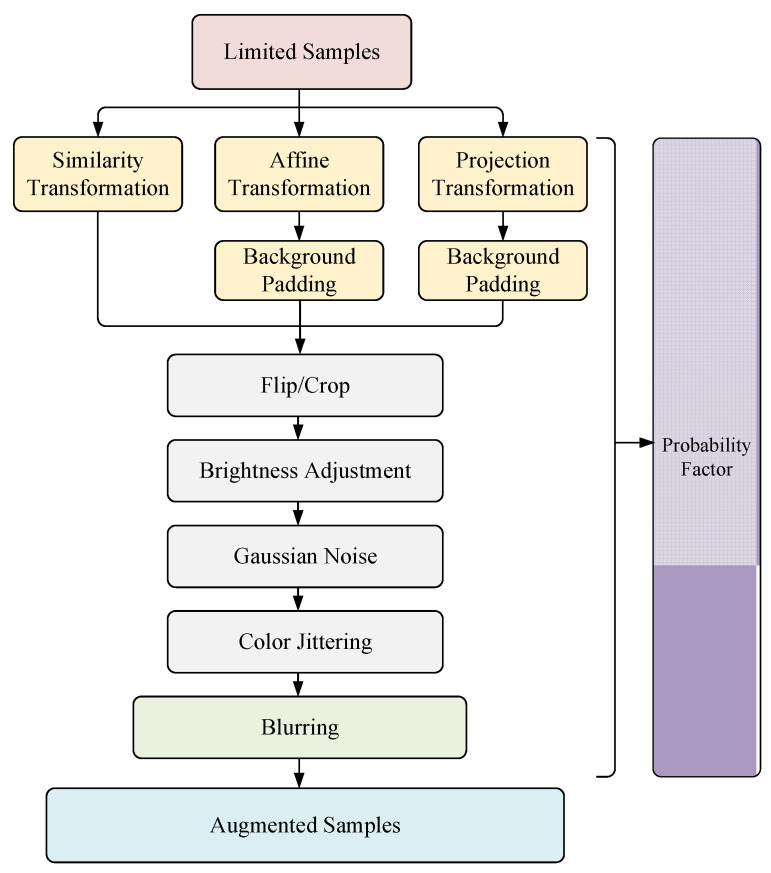
Framework of Limited Samples Augmentation.

We designed a complete set of limited samples augmented model, as shown in Figure 2 below. Firstly, three transformations including similarity, affine, and projection are carried out on limited samples to deal with possible rotation and deformation of samples in the 3D space. Secondly, the normal augmentation modules such as flip, crop, brightness adjustment, gaussian noise, and color Jittering will be implemented through transforming the augmented samples. Finally, a blurring augmentation is designed to deal with image blurring problems caused by visual platform jitter and high-speed objects. Besides, for the conventional augmentation module and blurring module, we provide a probability model to guarantee the random diversity of the augmentation module and realize the simulation under various interference combinations.

### 3.2. Transformations

This paper will not describe commonly used augmentation methods, such as flip, Crop, Gaussian noise, and color Jittering. Common image augmentation methods such as optical distortion, motion blur, and elastic transform, etc. can be implemented in the image augmentation library Albumentations [28]. This section will mainly explain the methods of affine transformation and projection transformation and give corresponding examples.

#### 3.2.1. Affine Augmentation

The algebraic definition of the affine transformation is as follows: the transformation from $R^{2}$ to itself is f, and if the relation between any vector $v\left(x,y\right)\subseteq R^{2}$ and its mapping relations $f\left(v\right)=\dot{v}\left(\dot{x},\dot{y}\right)$ is determined by Formula (1), then f is called the affine transformation in $R^{2}$.

(1) $$\{\begin{array}{l} x^{\prime }=a_{11}x+a_{12}y+a_{13}\\ y^{\prime }=a_{21}x+a_{22}y+a_{23} \end{array},\left|\begin{array}{cc} a_{11} & a_{12}\\ a_{21} & a_{22} \end{array}\right|\neq 0$$

According to Formula (1), the affine transformation from any vector v(x,y)  to $\dot{v}\left(\dot{x},\dot{y}\right)$ on the two-dimensional plane can be divided into a linear transformation and a translation, that is, multiplied by a matrix and plus a vector:

(2) $$\dot{v}=Av+b$$

where $A=\left|\begin{array}{cc} a_{11} & a_{12}\\ a_{21} & a_{22} \end{array}\right|$; $b=\left|\begin{array}{c} a_{13}\\ a_{23} \end{array}\right|$.

**Figure 3:**
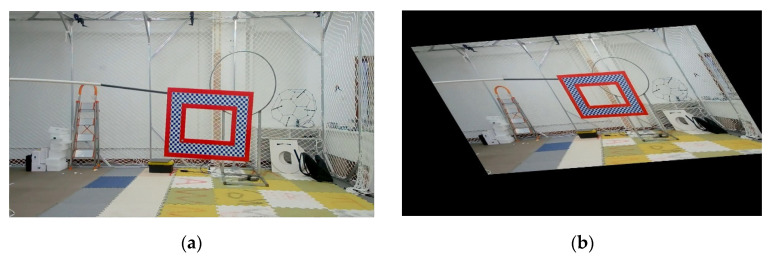
An example of affine transformation of samples in the OTPM dataset: (**a**) Before the transformation; (**b**) After the transformation.

According to the set definition of affine transformation, an affine transformation has the following properties: (1) maintain the flatness of two-dimensional graphics, and collinear points remain collinear points after affine transformation; (2) keep the parallelism of the graph, and the parallel line is still straight after affine transformation, but the included angle of the vector may change; (3) keep the simple ratio of three collinear points, that is, keep the ratio of two parallel line segments unchanged. In visual detection, as there are more or less geometric changes in both the image to be checked and the reference image, we simulate such changes through affine transformations. The rotation range of the affine transformation set in the experiment of this article is [0,45°] Figure 3 below shows the sample before and after affine transformation in the OTPM dataset.

#### 3.2.2. Projection Augmentation

**Figure 4:**
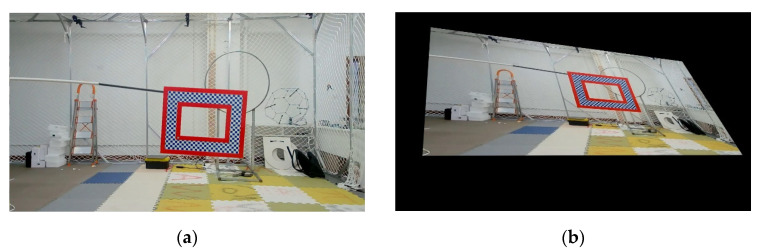
An example of affine transformation of samples in the OTPM dataset: (**a**) Before the transformation; (**b**) After the transformation.

Projection transformation, also known as perspective transformation, is essentially a process in which every point on the plane $P^{\prime }$ is projected onto the plane P under the action of perspective. If the plane P is defined as the plane of the object’s frontal view, the projection transformation is a process in which every pixel on the plane is transformed to the corresponding pixel on the frontal view. The projection transformation formula of the image is as follows:

(3) $$u=\frac{ax+by+c}{gx+hy+1}$$

(4) $$v=\frac{dx+ey+f}{gx+hy+1}$$

where (x,y) is the coordinate of the projected image, u,v is the coordinate of the original image, and (a,b,c,d,e,f,g,h) corresponds to the distortion parameters. Projection transformation widely exists in the three-dimensional empty object tracking problems. Due to camera angle change or object space rotation, projection transformation effect will be produced in the two-dimensional plane of the camera. The adaptability of the model to this kind of scene can be greatly increased by using projection transformation. The rotation range of the projected transformation set in the experiment of this article is [0,45°]. Figure 4 below shows the sample before and after affine transformation in the OTPM dataset.

### 3.3. Background Padding for Affine and Projection

**Figure 5:**
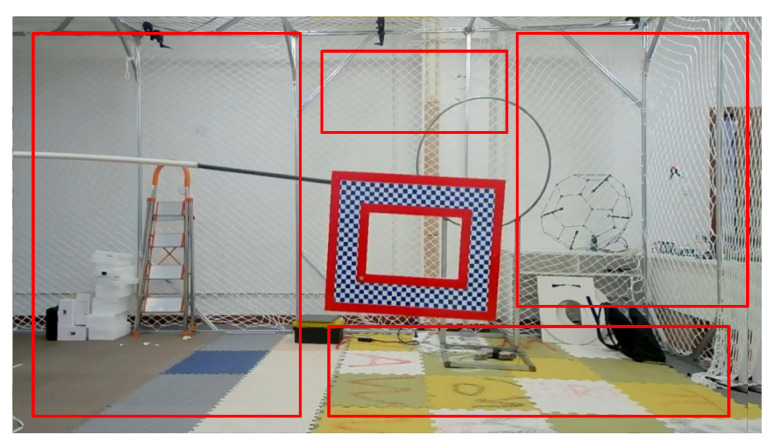
Random selection of non-object areas: The black background is supplemented with the randomly selected background shown in the red frame, which ensures the similarity of the background area and is beneficial to the training of the model on negative samples.

As shown in Figure 3 and Figure 4 in Section 3.2, after affine transformation and projection transformation are used, a black background will appear in the enlarged image. To prevent problems such as over-fitting, random clipping non-object areas are used to fill up the black area. Experiment 3 in Section 4 shows that the filled sample set is helpful to object detection performance. The specific padding formula is shown in the figure below:

(5) $$I\left(x,y\right)=\{\begin{array}{c} g\left(x,y\right),if\;t\left(x,y\right)=0\\ t\left(x,y\right),if\;t\left(x,y\right)\neq 0 \end{array}$$

where I(x,y) is the image after padding, g(x,y) is the background image, and t(x,y) is the image after affine or projection. Figure 5 shows the random selection of non-object areas.

**Figure 6:**
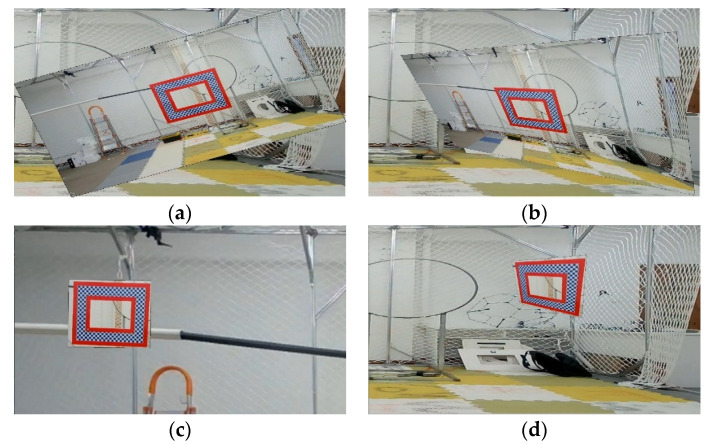
Schematic diagram of background padding: (**a**,**b**) background padding in first case; (**c**,**d**) background padding in second case.

As for the background padding problem, we consider it in two cases: the first case is that the geometric transformation area is the sample anchor box labeling area; the second case is that the geometric transformation area is the entire image. The first case is for the situation where the sample object is rotated, and the second case is for the situation where the viewing angle of the entire motion platform changes. Figure 6 shows the padding schematic for the two cases.

### 3.4. Blurring Augmentation

**Figure 7:**
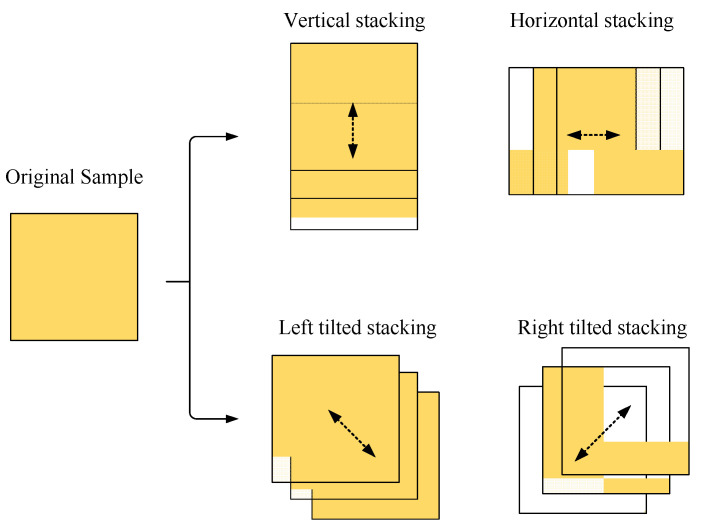
Multi-directional overlay blurring algorithm.

Three main factors that cause the blur are camera shake, fast-moving object, and focusing error. Among them, common image blur algorithms, such as mean blur and Gaussian blur, focus on the original object and operate with a blur convolution kernel. This method can effectively simulate the blur of focusing error. However, due to the blur caused by the camera shake and the fast movement of the object, especially when using the shutter door camera imaging, the expression form on the image is often the displacement and superposition of multiple images. Therefore, we design a multi-directional overlay blur algorithm to simulate the situation mentioned above, and the specific implementation is shown in Figure 7 below:

As shown in the above figure, a multi-directional overlay method is adopted to simulate the motion blur situation. After the transparency processing of the original sample, superposition operations are carried out in eight directions, including the vertical direction, horizontal direction, 45 degrees left, and 45 degrees right. The key parameters of the algorithm are designed, which n is the numbers of each superposition; $t_{b}$ is the moving step in the corresponding direction; $\alpha_{bi}$ is the transparency of each superimposed sample, i=1,2,3…n, and its value is [0,1], 1 is completely transparent, and 0 is completely opaque. Using the RGB image model as an example, the blurring algorithm formula is defined below:

(6) $$R\left(p_{A}\right)=\overset{n}{\underset{i=1}{\sum }}T_{i}(R\left(p_{i}\right)\cdot \alpha_{bi})\;i=1,2,3\ldots n$$

(7) $$G\left(p_{A}\right)=\overset{n}{\underset{i=1}{\sum }}T_{i}(G\left(p_{i}\right)\cdot \alpha_{bi})\;i=1,2,3\ldots n$$

(8) $$B\left(p_{A}\right)=\overset{n}{\underset{i=1}{\sum }}T_{i}(B\left(p_{i}\right)\cdot \alpha_{bi})\;i=1,2,3\ldots n$$

(9) $$T_{i}=\left[\begin{array}{ccc} 1 & 0 & t_{ui}\\ 0 & 1 & t_{vi}\\ 0 & 0 & 1 \end{array}\right]\;i=1,2,3\ldots n$$

**Figure 8:**
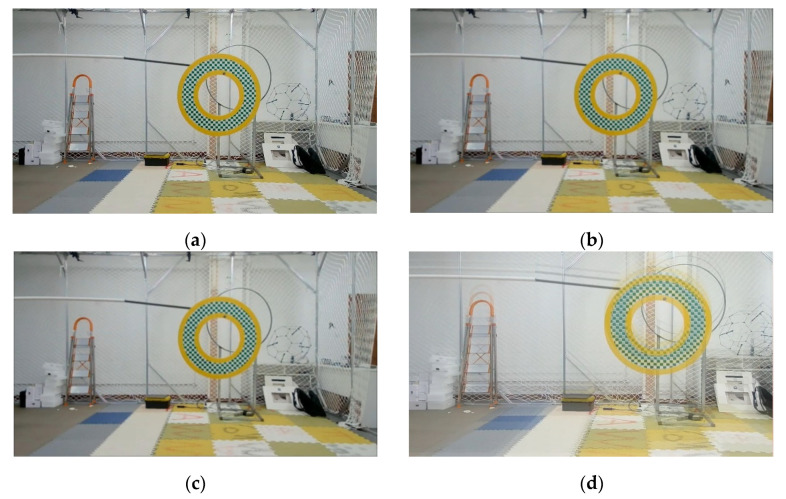

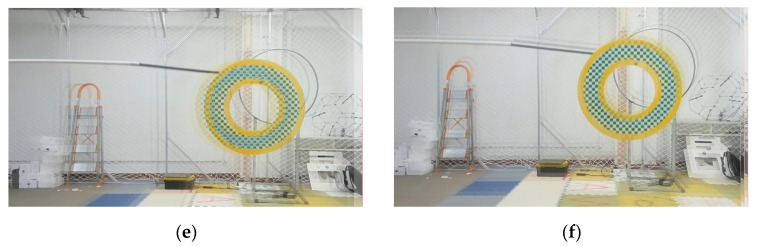
Schematic diagram of blurring augmentation: (**a**) sample before augmentation; (**b**) Gaussian blur, the size of the convolution kernel is 5 × 5; (**c**) The median blurring, the size of the convolution kernel is 5 × 5; (**d**–**f**) Multi-directional overlay blurring, *n* = 3, $t_{b}=\mathrm{20},a_{bi}=0.33,i=1,2,3$, the stacking directions are upward, rightward, and 45° rightward respectively.

The $p_{A}$ represents the pixel value of the corresponding position of the sample after image augmentation. According to Formulas (6)–(8), the pixel value of each channel is composed of the pixel displacement and overlay of the sample pixel after increasing the transparency. $T_{i}$ is the displacement matrix of the corresponding superposition subgraph, where $t_{ui}$ and $t_{vi}$ are the displacement value in the image coordinate system and jointly determined by the superposition direction, superposition step $t_{b}$ and superposition number n:

(10) $$t_{ui}=i\cdot t_{b}\cdot \delta_{u}$$

(11) $$t_{vi}=i\cdot t_{b}\cdot \delta_{v}$$

where $\delta_{u}$ and $\delta_{v}$ represent whether there is displacement in the augmentation direction. If there is no displacement, it is 0; if there is displacement, it is 1. After the Augmentation, the image area will increase, and the edge part of the image need to be cut to keep an appropriate size. Besides, two commonly used blur algorithms, median blur and Gaussian blur, are retained in our augmentation model. Figure 8 below is a schematic diagram of blurring augmentation OTMP sample set for blurring augmentation:

### 3.5. Probability Factors

The Probability Factor refers to the idea of the SSD model, that is, the randomness of samples can be improved by increasing the randomness of the augmented module combinations. In SSD, the probability of each module is estimated to be fixed at 0.5, while in our model, the Probability Factor is different and variable for each module, and its value range is [0,1]. For the regular augmented module and the blurring module, we give the corresponding probability factor $\eta_{i}$, and i is for different augmented modules. That is to say, for each augmentation, the augmented module has a probability of $\eta_{i}$ to carry out the augmentation operation, and a probability of $1-\eta_{i}$ to skip the augmented module. Meanwhile, the probability factor can also control the quantity of sample augmentation to prevent the training burden caused by too many samples.

In different task scenarios, researchers can adjust the probability factors of each module according to different visual scenarios. We give the reference values of 5 groups of probability factors for different scenarios in Table 2 below:

Table 2 above gives the proposed combination of probability factors in different object tracking scenarios. Among them, No. 1 is the recommended value for the unknown environment, and it could be used for visual tasks with unclear environmental characteristics and sample augmentation in advance. It can be seen from the bold probability factor values in the scenes numbered 2–5 that for objects with obvious rotation, we recommended increasing the probability factor of geometric augmentation. For objects with occlusion, we recommended increasing the probability factor of crop augmentation. For outdoor objects, we recommended increasing the probability factor of geometric augmentation. For long-time tracking and unstable illumination, we recommended increasing the probability factor of brightness augmentation. For platform jitter and high-speed objects, we recommended increasing the probability factor of blur augmentation.

### 3.6. Framework for Object Tracking Based on Motion Platform

**Figure 9:**
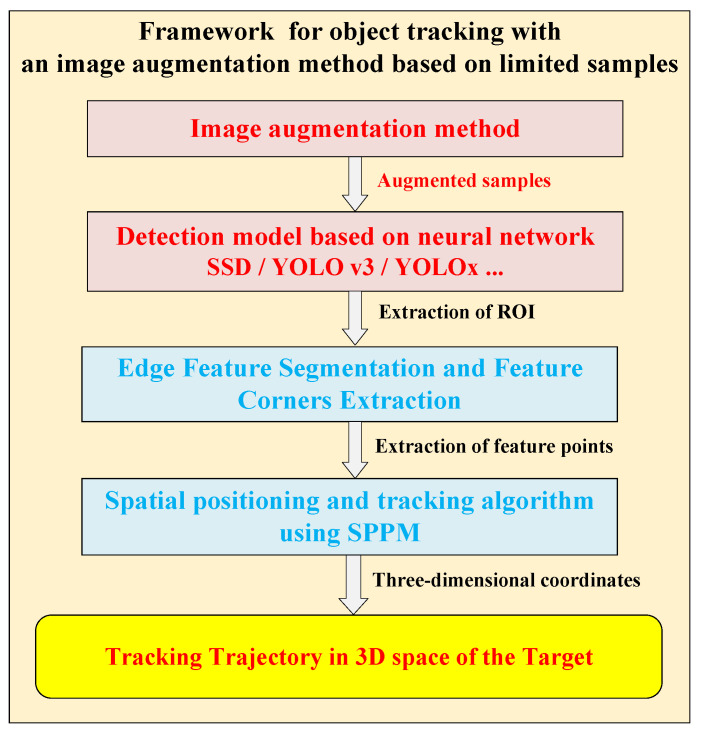
Framework for object tracking with our augmentation method.

Figure 9 shows the 3D object tracking algorithm based on the our augmentation method.

The 3D object tracking framework can be used for non-cooperative object tracking tasks with only a small number of samples. The main parts of the framework are as follows:

1. Image augmentation module: Based on a small amount of sample sets, affine transformation and projection transformation are firstly used to simulate the rotation of the object in the frame. Then the multi-direction blur algorithm is used to simulate the blurring caused by the jitter of the sensor platform and the high-speed object. Finally, the general augmentation model and augmentation probability factors are combined to realize the augmentation tasks of the sample;
2. Neural network detection model: The augmented samples are put into the neural network model for training to obtain the network parameters and extract the ROI anchor frame area of the object. This experiment adopts SSD and YOLOv3 models;
3. Object feature segmentation and keypoints extraction algorithm: According to the needs of different object types and tracking tasks, the objects in the ROI are further segmented and extracted to obtain the required features. The neural networks at the level of semantic segmentation, such as U-Net [29], or traditional image processing methods can be adopted;
4. Three-dimensional positioning algorithm: The SPPM method is adopted to quickly solve the depth value of the object feature point, in order to obtain the spatial coordinates of the object and finally acquire the spatial trajectory information of the object. In the author’s previous work [19,20,30,31], we discuss in detail object feature extraction and detection (FDA-SSD), planar feature moving object localization method (SPPM), and an autonomous localization method for motion platforms based on object tracking (MLSS-VO) respectively. In order not to distract the reader, we will not elaborate on what has been published in the manuscript, instead, we will focus on the elaboration of the image augmentation method. Therefore, the correlation between object tracking and SLAM can refer to the author’s previous research results.

## 4. Experiments

Based on multiple sets of experiments, a quantitative analysis was carried out on the performance of the augmented model proposed in this paper. At the same time, an object tracking algorithm was combined to test and track the performance of the object tracking framework using the augmented model, followed by quantitative analysis. The data sets used in the experiment include OTB50 [21] and OTB100 [22], and a set of open-sourced Benchmark for Object Tracking with Motion Parameters (OTMP) designed by us [20]. In addition, the detection models applied in the experiment are SSD, YOLOv3, YOLOv4, and YOLOx [9,11,24,25]. It is worth noting that our training sample set is obtained by augmenting the first 3–5 images in the dataset. In the test part, we selected the subsequent pictures of the corresponding dataset as test data. Therefore, training data and test data are separated.

The parameters we used in our experiments are:

(1) The angular range of geometric transformation augmentation θ⊆(0,45°).
(2) The number of stacks that controls the multi-directional stack blur augmentation degree i = 3; pixel step size $t_{b}=20$; transparency parameter $\alpha_{bi}=0.33$.
(3) The cropping size $S_{padding}$ for random background padding, we recommend using a background with a side length greater than 100 pixels and an aspect ratio close to the original image ratio.
(4) Probabilistic augmentation factor $\eta_{i}$, since we are discussing augmentation methods for small sample sets. The selection of the probability factor depends on the characteristic environment. In Section 3.5, we supplement the value suggestion of the probability factor.

**Figure 10:**
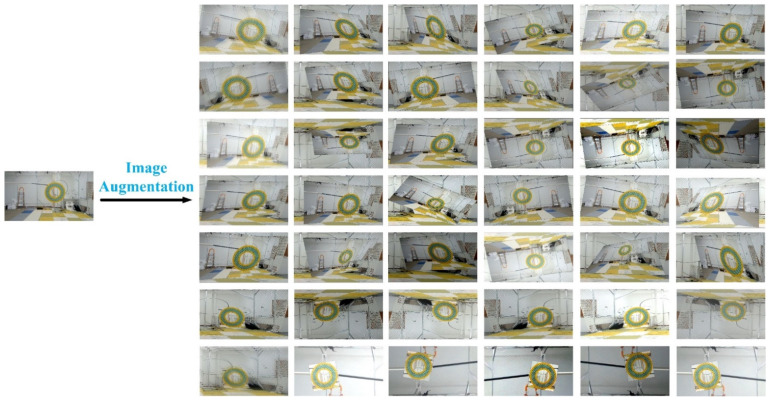
An augmentation example based on the OTMP test data set.

**Figure 11:**
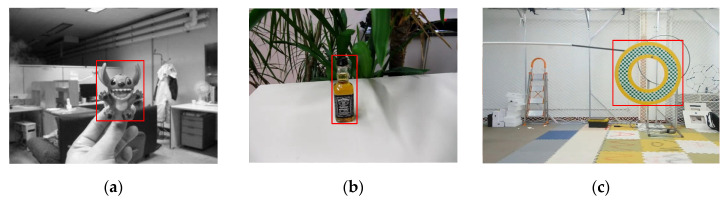
An example of the initial sample to be augmented: (**a**) OTB100-Toy, (**b**) OTB50-Liquor, (**c**) OTMP-Grid-like Circle.

**Experiment 1.** This experiment is to verify the effectiveness of the augmentation algorithm we proposed. We selected test subsets featured by spatial rotation, c or scale transformation in the OTB50, OTB100, and OTMP datasets, selected a small amount of 3–5 samples in each group as the initial samples to simulate the case of sample shortage, and applied the augmented model to increase the number of samples to 1000. Figure 10 shows an example of one OTMP sample expanded to 42 samples through our augmentation algorithm. The samples were put into SSD, YOLOv3, YOLOv4, and YOLOx for testing and experimental analysis. We compared the detection accuracy of the detection model without the augmentation method in this paper and the detection model with the augmentation model in this paper. Figure 11 shows an example of the initial sample we selected in Experiment 1.

Table 3 shows the accuracy comparison between the SSD, YOLOv3, YOLOv4, and YOLOx of the above datasets before and after augmentation.

The results in Table 3 indicate that the augmented model proposed in this paper can help the detection model to improve the accuracy at about least 10% under the condition of only a few samples available for model training. In particular, our model can improve tracking performance by nearly 15% for the object with planar characteristics such as the OTMP-Grid-like Circle. It can be seen that although the detection models of YOLOv3 and YOLOx come with new hybrid augmentation methods such as Mixup, Cutmix, and Mosaic. However, the augmented model in this paper can still effectively help the detection model to improve the object tracking problem with object rotation and platform shaking. Overall, the performance of YOLOv4 and YOLOx were slightly higher than that of YOLOv3 and SSD.

**Figure 12:**
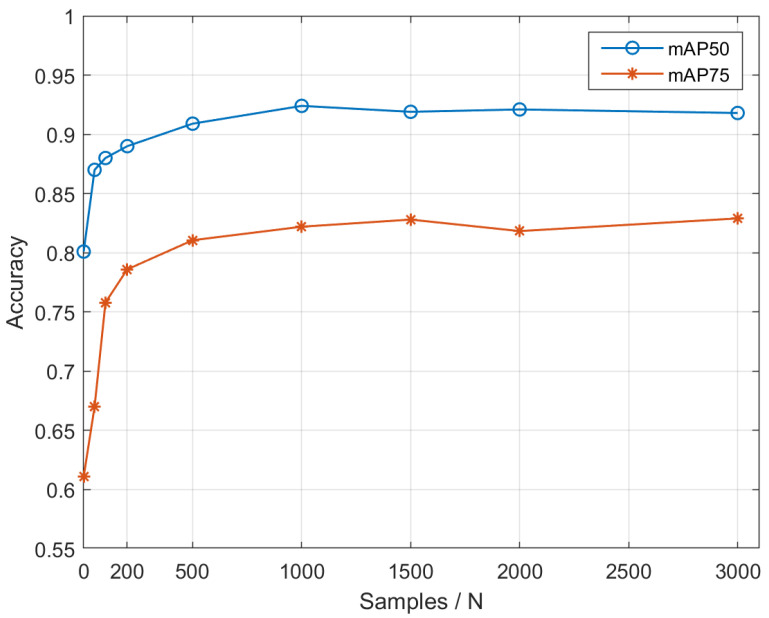
Schematic diagram of mAP50 and mAP75.

Further, we discuss the effect of the number of augmentations employed in our augmentation method on detection accuracy. We use YOLOv4 as the basic detection model, and compare the difference in detection accuracy between the model trained directly using 10 original samples and the model trained after sample augmentation using the augmentation method in this paper. The test dataset is OTMP-Grid-like Circle. Among them, for the experimental group using the augmented model in this paper, we set the augmentation number to 50, 100, 200, 500, 1000, 1500, 2000, 3000 respectively, and analyze the influence of the augmentation number on the accuracy. The following Figure 12 is a schematic diagram of mAP50 and mAP75 in the experiment.

It can be seen from Figure 12, for the OTMP dataset, when the samples exceed to 500, the augmentation module’s effect of improving the accuracy becomes relatively small. Therefore, it is an appropriate value to choose the augmentation number as 1000.

**Experiment 2.** This experiment is aimed at analyzing the effectiveness of the affine and projection augmentation methods applied in this paper. Similarly, we also used OTB and OTMP for augmentation and put them into SSD, YOLOv3, YOLOv4, and YOLOx for training. The augmentation number is 1000. The difference is that only two sub-modules such as affine and projection augmentation were blocked in the test group, and the others were still augmented. For the comparison group, the test results of the augmented data set in Table 1 can be a reference. The schematic diagram of the experiment is shown in Figure 3 and Figure 4 above, and the results of the comparative experiment are shown in Table 4.

From Table 3 and Table 4 it can be concluded that that affine and projection augmentation play a decisive role in improving object detection accuracy in the data set with rotation characteristics. Based on an analysis from another perspective, it can be concluded that traditional similarity transformation augmentation is not enough to cope with the complicated perspective transformation and the spatial rotation of the object itself in 3D object tracking. Therefore, it is necessary to introduce affine and projection augmentation in spatial tracking tasks based on motion platforms.

**Experiment 3.** This experiment mainly focuses on analyzing the effectiveness of the background padding method used in this paper. The augmentation number is 1000. The background padding module was blocked in the test group, and the others were still augmented. For the comparison group, the test results of the augmented data set in Table 1 can be a reference. The schematic diagram of the experiment is shown in Figure 6 above, and the results of the comparative experiment are shown in Table 5.

From Table 5 it can be concluded that the application of the background padding module can improve the accuracy of the detection model by about 0.5–2%, although the improvement is limited. Especially, the resulting black area became larger when the large-angle transformation happened in the samples, we still recommended using background padding for background supplementation.

**Experiment 4.** This experiment is aimed at analyzing the effectiveness of the multi-directional overlay blurring algorithm used in this paper. The augmentation number is 1000. The multi-directional overlay blurring module was blocked in the test group. For the comparison group, the test results of the augmented data set in Table 1 can be a reference. The schematic diagram of the experiment is shown in Figure 8 above, and the results of the Experiment 3 are shown in Table 6.

From Table 6 it can be concluded that the detection accuracy of scenes with motion blur using the multi-directional overlay blurring module is improved by about 5–9%, indicating that the traditional blur algorithm has limited ability to simulate the blur caused by the shaking of motion platforms, and the multi-directional overlay blurring is more effective to simulate real blurred scenes.

**Figure 13:**
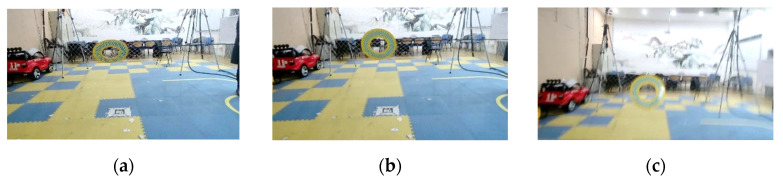
Examples of the moving objects to be tracked in OTMP-Grid-like Circle: (**a**–**c**) Dynamic sample sequence schematic.

**Experiment 5.** This experiment is to investigate the object tracking performance of the Framework for object tracking based on the mobile platform proposed by the augmented model in this paper. The Grid-like Circle data set in OTMP was used, which provided the trajectory of the sensor platform and the object in the indoor environment and effectively verify the performance of the algorithm in this paper. Figure 12 shows an example of a moving object in the Grid-like Circle, and Figure 13 shows a comparison between the trajectory result estimated by the object tracking frame and the ground truth.

**Figure 14:**
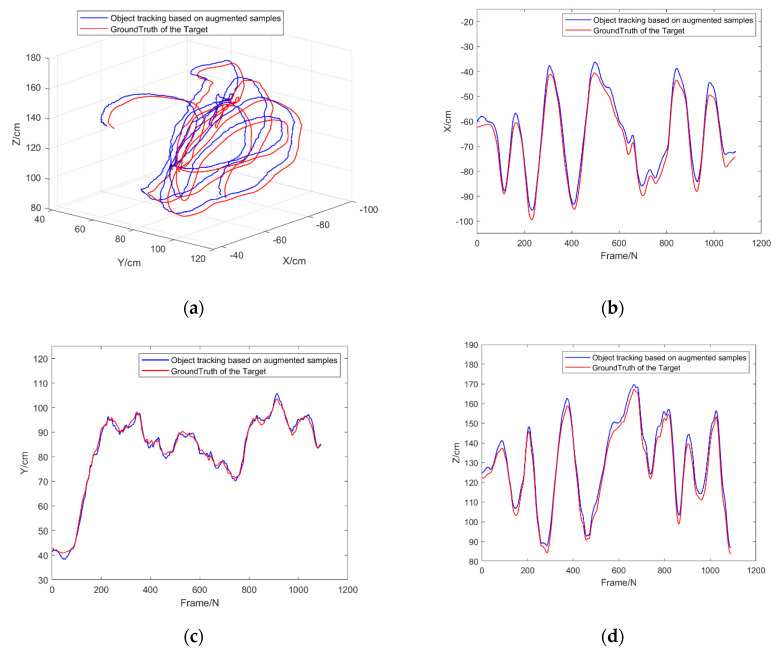
Comparison of estimated trajectory and Ground Truth of experiment 5: (**a**) Comparison of motion trajectories in Cartesian coordinate system; (**b**) Trajectory comparison in *X*-axis; (**c**) Trajectory comparison in *Y*-axis; (**d**) Trajectory comparison in *Z*-axis.

Table 7 shows the root mean square error of dynamic object tracking in experiment 5:

According to Figure 14 and Table 7, it can be concluded that the object tracking framework based on the augmented method in this paper can effectively deal with the problems such as the spatial rotation of moving objects and the image blur caused by jitter. The RMSE for tracking three-dimensional objects is less than 4.21 cm, indicating that the demands of most indoor tracking applications can be fulfilled.

## 5. Conclusions

An Image Augmentation Method Based on Limited Samples for Object Tracking based on the Mobile Platform is proposed in this paper to achieve effective tracking of the frame moving objects. Our method is aiming at solving the problem of the insufficient generalization ability of neural networks when training a small number of samples under the background of object tracking. The augmented model used three geometric transformations such as similarity, affine, and projection to deal with complex spatial vision transformations. In addition, a multi-directional overlay blurring augmentation method was proposed to solve the image blur caused by sensor jitter, and a complete set of Limited Samples augmented model was constructed by combining with conventional augmentation methods and augmentation probability factors. Based on this model, we proposed a Framework for object tracking based on the motion platform.

Multiple groups of experiments have verified: (1) The augmented module proposed in this paper is conducive for the detection model to improve the detection accuracy by at least 10% when only a few samples are available for model training and spatial rotation occurs in moving objects. Especially for objects with planar characteristics, the affine and projection transformation used in this paper can greatly improve the detection accuracy of the model; (2) The background padding module proposed in this paper can improve the accuracy of the detection model by about 0.5–2%; (3) The multi-directional overlay blurring method proposed in this paper performed great in simulating the image blur caused by platform jitter and fast motion, and it is able to improve the detection accuracy by about 5–9% in the experiment; (4) Based on the object tracking framework of this augmented model, the RMSE is estimated to be less than 4.21 cm in terms of the actual tracking of indoor 3D objects, indicating it is applicable to most tasks of indoor object tracking.

The method proposed in our paper is suitable for most kinds of motion vision platforms such as unmanned aerial vehicles, unmanned vehicles, and industrial inspection platforms. Future research directions include: (1) Autonomously learn augmentation probability factors in different visual tracking tasks to obtain a more effective augmentation sample set. (2) The main limitation of our method is that since the augmentation method in this paper is aimed at scenarios with limited initial samples, some parameters of the augmented model used in this paper depend on the user’s experience settings. Therefore, after obtaining the initial tracking frame, how to extract new samples online and adjust the online augmentation strategy autonomously is a problem worthy of further research by posterior researchers.

## Figures and Tables

**Table 1:** Comparison between our Augmentation model and Augmentation based on general model.

Augmentations	Building Method	Advantages	Disadvantages
Augmentation based on general detection model	Use augmented modules in existing detection models (SSD, Yolo v4, Yolo x, etc.)	End-to-end augmentation; no redundant design required.	Augmentation may not be ideal in certain scenarios; detection scenarios with insufficient samples are not applicable.
Our Augmentation framework	A suitable augmented model is constructed for the needs of object rotation and imaging blur in the mobile platform.	Targeted for object tracking scenarios; Suitable for scenarios with fewer pre-training samples.	The construction of the augmented model needs to analyze the characteristics of the scene; the construction process is relatively cumbersome.

**Table 2:** Reference value of probability factor.

No.	Scenarios	Geometric Transformation	Brightness Adjustment	Flip	Crop	Gaussian Noise	Color Jittering	Burring
1	Unknown scene	0.5	0.5	0.5	0.2	0.2	0.2	0.5
2	Indoor/Object rotation/Platform shake	0.8	0.1	0.5	0.1	0.1	0.1	0.8
3	Indoor/Object occlusion/High-speed moving object	0.5	0.1	0.5	0.6	0.1	0.1	0.8
4	Outdoor short-term tracking/No rotation/Platform shaking	0.3	0.5	0.1	0.2	0.3	0.3	0.8
5	Outdoor long-term tracking/Object rotation/Fixed platform	0.8	0.8	0.5	0.2	0.3	0.3	0.4

**Table 3:** Comparison between the SSD, YOLOv3, YOLOv4, and YOLOx.

Methods	Datasets	Input Resolution	Augmented	mAP50	mAP75
SSD	OTB100-Toy	320 × 240	**Yes**	**0.729**	**0.583**
SSD	OTB100-Toy	320 × 240	No	0.608	0.510
SSD	OTMP-Grid-like Circle	1280 × 720	**Yes**	**0.893**	**0.781**
SSD	OTMP-Grid-like Circle	1280 × 720	No	0.596	0.472
YOLOv3	OTB100-Toy	320 × 240	**Yes**	**0.752**	**0.598**
YOLOv3	OTB100-Toy	320 × 240	No	0.648	0.521
YOLOv3	OTMP-Grid-like Circle	1280 × 720	**Yes**	**0.913**	**0.841**
YOLOv3	OTMP-Grid-like Circle	1280 × 720	No	0.692	0.575
YOLOv4	OTB100-Toy	320 × 240	**Yes**	**0.791**	**0.648**
YOLOv4	OTB100-Toy	320 × 240	No	0.672	0.560
YOLOv4	OTMP-Grid-like Circle	1280 × 720	**Yes**	**0.924**	**0.822**
YOLOv4	OTMP-Grid-like Circle	1280 × 720	No	0.801	0.611
YOLOx	OTB100-Toy	320 × 240	**Yes**	**0.789**	**0.638**
YOLOx	OTB100-Toy	320 × 240	No	0.633	0.564
YOLOx	OTMP-Grid-like Circle	1280 × 720	**Yes**	**0.936**	**0.851**
YOLOx	OTMP-Grid-like Circle	1280 × 720	No	0.812	0.632

**Table 4:** Performance comparison before and after the affine and projection augmentation.

Methods	Datasets	Input Resolution	Affine & Projection	mAP50	mAP75
SSD	OTB50-Liquor	640 × 480	**Yes**	**0.821**	**0.747**
SSD	OTB50-Liquor	640 × 480	No	0.737	0.622
SSD	OTMP-Grid-like Circle	1280 × 720	**Yes**	**0.893**	**0.781**
SSD	OTMP-Grid-like Circle	1280 × 720	No	0.640	0.517
YOLOv3	OTB50-Liquor	640 × 480	**Yes**	**0.837**	**0.737**
YOLOv3	OTB50-Liquor	640 × 480	No	0.778	0.672
YOLOv3	OTMP-Grid-like Circle	1280 × 720	**Yes**	**0.913**	**0.841**
YOLOv3	OTMP-Grid-like Circle	1280 × 720	No	0.602	0.512
YOLOv4	OTB50-Liquor	640 × 480	**Yes**	**0.886**	**0.780**
YOLOv4	OTB50-Liquor	640 × 480	No	0.792	0.705
YOLOv4	OTMP-Grid-like Circle	1280 × 720	**Yes**	**0.924**	**0.822**
YOLOv4	OTMP-Grid-like Circle	1280 × 720	No	0.840	0.657
YOLOx	OTB50-Liquor	640 × 480	**Yes**	**0.878**	**0.789**
YOLOx	OTB50-Liquor	640 × 480	No	0.784	0.712
YOLOx	OTMP-Grid-like Circle	1280 × 720	**Yes**	**0.936**	**0.851**
YOLOx	OTMP-Grid-like Circle	1280 × 720	No	0.839	0.660

**Table 5:** Performance comparison before and after the background padding augmentation.

Methods	Datasets	Input Resolution	Background Padding	mAP50	mAP75
SSD	OTB50-Liquor	640 × 480	**Yes**	**0.821**	**0.747**
SSD	OTB50-Liquor	640 × 480	No	0.817	0.728
SSD	OTMP-Grid-like Circle	1280 × 720	**Yes**	**0.893**	**0.781**
SSD	OTMP-Grid-like Circle	1280 × 720	No	0.870	0.785
YOLOv3	OTB50-Liquor	640 × 480	**Yes**	**0.837**	**0.737**
YOLOv3	OTB50-Liquor	640 × 480	No	0.815	0.712
YOLOv3	OTMP-Grid-like Circle	1280 × 720	**Yes**	**0.913**	**0.841**
YOLOv3	OTMP-Grid-like Circle	1280 × 720	No	0.901	0.838
YOLOv4	OTB50-Liquor	640 × 480	**Yes**	**0.886**	**0.780**
YOLOv4	OTB50-Liquor	640 × 480	No	0.872	0.778
YOLOv4	OTMP-Grid-like Circle	1280 × 720	**Yes**	**0.924**	**0.822**
YOLOv4	OTMP-Grid-like Circle	1280 × 720	No	0.915	0.817
YOLOx	OTB50-Liquor	640 × 480	**Yes**	**0.878**	**0.789**
YOLOx	OTB50-Liquor	640 × 480	No	0.851	0.768
YOLOx	OTMP-Grid-like Circle	1280 × 720	**Yes**	**0.936**	**0.851**
YOLOx	OTMP-Grid-like Circle	1280 × 720	No	0.932	0.847

**Table 6:** Comparison before and after the multi-directional overlay blurring augmentation.

Methods	Datasets	Input Resolution	Blurring	mAP50	mAP75
SSD	OTB50-BlurOwl	640 × 480	**Yes**	**0.731**	**0.649**
SSD	OTB50-BlurOwl	640 × 480	No	0.610	0.508
SSD	OTMP-Grid-like Circle	1280 × 720	**Yes**	**0.893**	**0.781**
SSD	OTMP-Grid-like Circle	1280 × 720	No	0.801	0.714
YOLOv3	OTB50-BlurOwl	640 × 480	**Yes**	**0.726**	**0.660**
YOLOv3	OTB50-BlurOwl	640 × 480	No	0.636	0.462
YOLOv3	OTMP-Grid-like Circle	1280 × 720	**Yes**	**0.913**	**0.841**
YOLOv3	OTMP-Grid-like Circle	1280 × 720	No	0.821	0.730
YOLOv4	OTB50-BlurOwl	320 × 240	**Yes**	**0.782**	**0.629**
YOLOv4	OTB50-BlurOwl	320 × 240	No	0.712	0.554
YOLOv4	OTMP-Grid-like Circle	1280 × 720	**Yes**	**0.924**	**0.822**
YOLOv4	OTMP-Grid-like Circle	1280 × 720	No	0.873	0.766
YOLOx	OTB50-BlurOwl	320 × 240	**Yes**	**0.802**	**0.673**
YOLOx	OTB50-BlurOwl	320 × 240	No	0.743	0.630
YOLOx	OTMP-Grid-like Circle	1280 × 720	**Yes**	**0.936**	**0.851**
YOLOx	OTMP-Grid-like Circle	1280 × 720	No	0.878	0.807

**Table 7:** RMSE of object tracking in Experiment 5.

Axis	*X* [cm]	*Y* [cm]	*Z* [cm]
RMSE	4.15	3.18	4.21

## Data Availability

The complete dataset of Benchmarks for Object Tracking with Motion Parameters (OTMP) is available at GitHub: https://github.com/6wa-car/OTMP-DataSet.git.(accessed on 30 December 2021).
